# Synergistic Activity of Equol and Meropenem against Carbapenem-Resistant *Escherichia coli*

**DOI:** 10.3390/antibiotics10020161

**Published:** 2021-02-05

**Authors:** Hye-Rim Kim, Yong-Bin Eom

**Affiliations:** 1 Department of Medical Sciences, Graduate School, Soonchunhyang University, Asan 31538, Korea; goa6471@naver.com; 2 Department of Biomedical Laboratory Science, College of Medical Sciences, Soonchunhyang University, Asan 31538, Korea

**Keywords:** equol, carbapenem-resistant *Escherichia coli*, anti-bacterial, anti-virulence, synergistic activity

## Abstract

The emergence of carbapenem-resistant *Enterobacterales* (CRE) seriously limits treatment options for bacterial infections. Combined drugs are an effective strategy to treat these resistant strains. This study aimed to evaluate the synergistic effect of equol and meropenem against carbapenem-resistant *Escherichia coli*. First, this study investigated the antibacterial activity of carbapenems on clinically isolated *E. coli* strains by analyzing the minimum inhibitory concentrations (MICs). The *E. coli* strains were all resistant to carbapenem antibiotics. Therefore, we confirmed the cause of carbapenem resistance by detecting *bla*_KPC_ and *bla*_OXA-48_ among the carbapenemase genes using polymerase chain reaction (PCR) analysis. Checkerboard and time-kill analyses confirmed that equol restored the susceptibility of carbapenem-resistant *E. coli* to meropenem. Also, the transcription levels of specific carbapenemase genes in *E. coli* were significantly suppressed by equol. The study also evaluated the anti-virulence effects of equol on bacterial biofilm and motility through phenotypic and genotypic analyses. In conclusion, our results revealed that equol had a synergistic effect with meropenem on carbapenem-resistant *E. coli*. Therefore, this study suggests that equol is a promising antibiotic adjuvant that prevents the expression of carbapenemases and virulence factors in carbapenem-resistant *E. coli*.

## 1. Introduction

Carbapenems are antibiotics used to treat multidrug-resistant (MDR) bacterial infections as a last resort. Therefore, the emergence and rapid spread of carbapenem-resistant Enterobacterales (CRE) are serious, global public health problems, associated with high mortality and morbidity due to treatment failure [1]. Carbapenem resistance is mediated by several factors such as porin loss, overexpressed efflux pumps, and carbapenemase production [2]. Among these factors, previous studies have reported that the production of carbapenemases is the main mechanism of carbapenem resistance [3,4]. According to the Ambler classification, carbapenemases can be mainly divided into three classes, class A (KPC; *K. pneumoniae* carbapenemase), class B (NDM; New Delhi metallo-*β*-lactamase, IMP; imipenem-resistant *Pseudomonas* carbapenemase and Verona integron-encoded metallo-*β*-lactamase (VIM)), and class D (OXA-48; oxacillinase-48) [3,5].

*Escherichia coli* belonging to the family Enterobacterales are symbiotic microorganisms commonly found in the gastrointestinal tract of warm-blooded organisms [6]. However, certain pathogenic strains can cause intestinal and extra-intestinal diseases such as urinary tract infections, meningitis, sepsis, and hemorrhagic colitis by damaging host cells through virulence factors [7]. The virulence factors of *E. coli* include flagella-mediated motility and curli fibers, which promote biofilm development and pathogenicity [8,9]. Biofilm, a community of sessile cells encased in extracellular polysaccharides self-produced by microorganisms, promotes resistance to antibiotics because it protects bacteria from antibiotics [10]. Previous studies have reported that biofilm formation can threaten human health by protecting microorganisms from the host immune system [11,12]. Moreover, these pathogenic *E. coli* strains also tend to acquire genes encoding extended-spectrum *β*-lactamases, carbapenemases, which hydrolyze or inactivate antibiotics, leading to antibiotic resistance [13]. A previous study showed that the resistance of *E. coli* to carbapenems, as well as to penicillins and cephalosporins, had increased [14]. 

The synergistic effect from the combination of two or more antibiotics may be an effective strategy to expand the antibacterial spectrum, minimize toxicity and prevent the emergence of MDR bacteria than when used alone [15]. Also, the combination of natural compounds and antibiotics is recommended to effectively treat MDR bacteria by minimizing adverse effects [16]. Many studies have reported synergistic effects from combinations of antibiotics and plant extracts [17,18,19]. Among them, a recent study found that the combination of antibiotics and natural phenolic compounds against *E. coli* showed synergistic effects [20]. Therefore, new effective compounds that can replace or synergize with existing antibiotics are urgently needed to treat carbapenem-resistant *E. coli* strains.

Isoflavone, a type of naturally occurring phenolic compound, is mainly sourced from soybeans, which contain daidzein, glycitein, and genistein [21]. In addition, isoflavone is called phytoestrogen because it is not only structurally similar to 17-*β*-estradiol but also beneficial to human health. Equol (4′,7-isoflavandiol) is a metabolite of daidzein formed by intestinal bacteria such as family Coriobacteriaceae and *Lactobacillus* sp. [22]. Previous studies demonstrated that equol exhibited more effective antioxidant and estrogenic activities than daidzein [23,24,25]. Moreover, many researchers have shown that equol had various biological activities such as anti-cancer, anti-inflammatory, and antimicrobial effects [26,27,28,29,30]. Although equol has biological activity, to the best of our knowledge, the anti-bacterial and anti-virulence effects of equol against carbapenem-resistant *E. coli* have not yet been reported.

This study assessed the synergistic and anti-virulence activities of equol against clinically isolated carbapenem-resistant *E. coli* strains. Also, this study investigated the mechanisms related to carbapenem-resistant *E. coli* and the synergistic effect of equol combined with meropenem.

## 2. Results

### 2.1. Detection of Carbapenem-Resistant E. coli

The MIC values of carbapenems (meropenem, imipenem, and ertapenem) for *E. coli* strains (KBN12P05816 and KBN12P06081) ranged from ≤4 to 64 μg/mL (Table 1). Specifically, *E. coli* strains were identically inhibited in terms of cell growth by 90% at 16 μg/mL of meropenem and imipenem ≥ 4 μg/mL. Also, *E. coli* strains (KBN12P05816 and KBN12P06081) were inhibited by 90% at 32 μg/mL and 64 μg/mL concentrations of ertapenem, respectively. Therefore, according to the Clinical and Laboratory Standards Institute (CLSI) breakpoints, the *E. coli* strains were identified as carbapenem-resistant strains. Since the isolates were resistant to carbapenems, PCR was used to check for the presence of five major carbapenemase genes. As a result, *bla*_KPC_ and *bla*_OXA-48_ were detected in the clinically isolated *E. coli* strains (Table 2).

### 2.2. Equol Restores the Susceptibility of Carbapenem-Resistant E. coli to Meropenem

**Figure 1:**
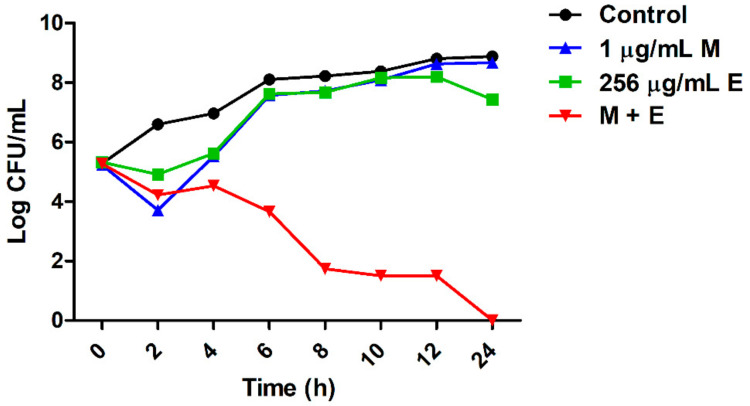
Synergistic effect of equol and meropenem against carbapenem-resistant *E. coli*. *E. coli* KBN12P05816 strain treated with equol and meropenem at 37 °C. Each sample was plated with a spreader on MHA and incubated at 37 °C for 24 h. The CFU/mL values were recorded. (●) Control; (■) 256 μg/mL of equol; (▲) 1 μg/mL of meropenem; (▼) the combination of 1 μg/mL of meropenem plus 256 μg/mL of equol. A sample not treated with equol and meropenem was used as the control.

To determine the synergistic effect of equol and meropenem on carbapenem-resistant *E. coli*, checkerboard and time-kill analyses were performed. The minimum inhibitory concentrations (MICs) for equol and meropenem against carbapenem-resistant *E. coli* strains (KBN12P05816 and KBN12P06081) are shown in Table 3. The MIC is the lowest concentration of antimicrobial agents that inhibited cell growth by 90% compared to the control. The MIC of equol and meropenem for the two *E. coli* strains were 1024 and 16 μg/mL, respectively. By measuring the absorbance, equol was found to inhibit the cell growth of *E. coli* in a concentration-dependent manner. When meropenem was combined with equol, the MIC of meropenem plus equol was 1 plus 256 μg/mL, respectively, against the *E. coli* strains. The fractional inhibitory concentration (FIC) and FIC index (FICI) were calculated as described in the methods and the FICI of the equol and meropenem combination for all *E. coli* strains was 0.31 (Table 3). FICI values of less than or equal to 0.5 indicate a synergistic effect. Therefore, the FICI value for the combination of meropenem plus equol indicated a synergistic effect against these carbapenem-resistant *E. coli* strains. The analysis of the synergistic effects of meropenem and equol on *E. coli* KBN12P05816 over time showed that only the combined compounds were able to kill carbapenem-resistant *E. coli*. The antibacterial activity of the combined compounds was more than 6 log_10_ compared to the control after 8 h (Figure 1).

### 2.3. Anti-Biofilm Effects of Equol against Carbapenem-Resistant E. coli

**Figure 2:**
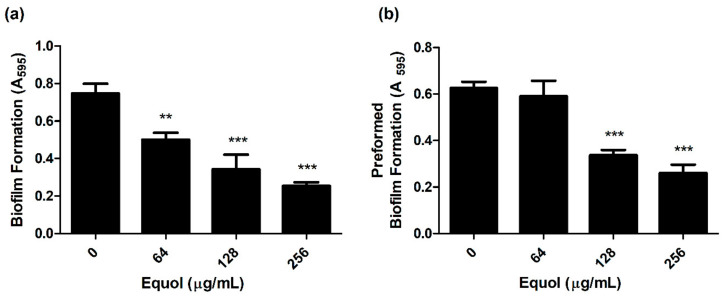
Anti-biofilm effect of equol against carbapenem-resistant *E. coli*. Biofilm inhibition (**a**) and biofilm eradication (**b**) of *E. coli* KBN12P05816 were quantified using crystal violet staining. *E. coli* was grown in TSB. The biofilm biomass was measured by the absorbance at 595 nm. Asterisks (*) indicate statistically significant differences (** *p* < 0.01, and *** *p* < 0.001) from the control.

To investigate whether equol inhibited the biofilm of carbapenem-resistant *E. coli*, the biofilm biomass was quantified by crystal violet staining. As shown in Figure 2a, equol inhibited the biofilm formation of *E. coli* in a concentration-dependent manner compared to the control. Equol not only showed an inhibitory effect on biofilm formation but also showed an effect on preformed biofilm in a concentration-dependent manner (Figure 2b). More specifically, 256 μg/mL equol inhibited the biofilm formation of *E. coli* by 79.3% and the preformed biofilm of *E. coli* was inhibited by 76.7% in the presence of 256 μg/mL equol.

### 2.4. Equol Suppresses the Motility of Carbapenem-Resistant E. coli

**Figure 3:**
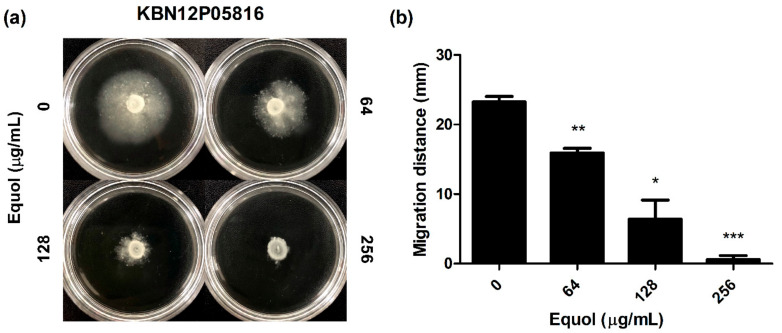
Effect of equol on the motility of *E. coli*. (**a**) Motility analyses were performed using semisolid agar plates containing equol at various concentrations. Carbapenem-resistant *E. coli* KBN12P05816 (1 × 10^6^ CFU/mL) was inoculated in the center of the semisolid agar containing equol. The inoculated plates were incubated at 37 °C for 24 h and (**b**) the migration distance (mm) was measured. The error bars represent the means and standard deviations (SDs). Asterisks (*) indicate statistically significant differences (* *p* < 0.05, ** *p* < 0.01 and *** *p* < 0.001) from the control.

The effect of equol on the motility of carbapenem-resistant *E. coli* was investigated as a phenotypic study. As shown in Figure 3, after an 18-h incubation, the migration distance was significantly decreased in semisolid agar plates containing equol in a dose-dependent manner (Figure 3a). Since the equol concentration used was at a sub-MIC, the decrease in motility was not due to impaired growth. In detail, the motility zones of *E. coli* treated with 64 and 128 μg/mL equol were 15.95 and 6.42 mm, which were 31.4 and 72.4% smaller than that of the dimethyl sulfoxide (DMSO)-treated control (Figure 3b). Moreover, when *E. coli* was exposed to 256 μg/mL equol, motility was hardly observed.

### 2.5. Equol Suppressed the Expression Levels of Carbapenemase- and Virulence-Related Genes in E. coli

**Figure 4:**
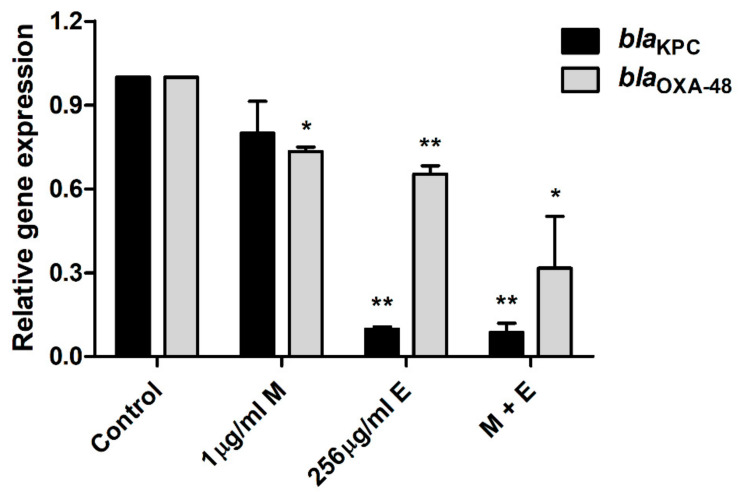
Transcriptional changes in the carbapenemase-related *E. coli* genes. After treatment with equol alone (256 μg/mL), meropenem alone (1 μg/mL), and the combination of 1 μg/mL of meropenem plus 256 μg/mL of equol, qPCR analysis of *bla*_KPC_ and *bla*_OXA-48_ genes in *E. coli* KBN12P05816 was performed. *16SrRNA* was used to normalize the transcriptional levels of the target genes. The expression levels of the genes are expressed as fold-changes compared to the control and analyzed using the Student’s *t*-test. The error bars represent the means and standard deviations (SDs). Asterisks (*) indicate statistically significant differences (* *p* < 0.05 and ** *p* < 0.01) from the control.

**Figure 5:**
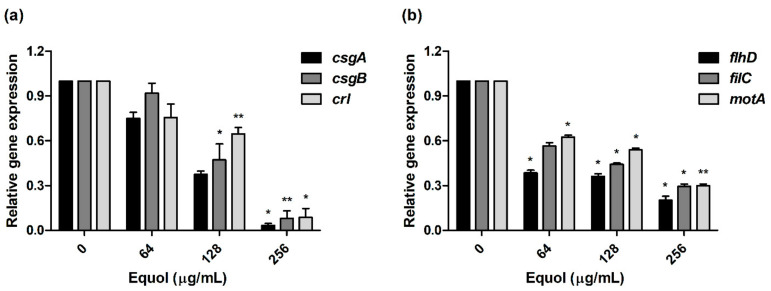
Transcriptional changes in the virulence-related *E. coli* genes. (**a**) Curli-related (*csgA*, *csgB,* and *crl*) genes and (**b**) motility-related (*fihD*, *filC,* and *motA*) genes. After treatment with various concentrations of equol (64 to 256 μg/mL), qPCR analysis of curli- and motility-related genes in *E. coli* KBN12P05816 was performed. The transcription levels of the target genes were normalized to that of *16S rRNA.* The Student’s *t*-test was conducted to analyze the gene expression between the samples and the control. The error bars represent the means and standard deviations (SDs). Asterisks (*) indicate statistically significant differences (* *p* < 0.05 and ** *p* < 0.01) from the control.

Quantitative polymerase chain reaction (qPCR) was performed to investigate the molecular mechanisms responsible for the carbapenem resistance of *E. coli* and the anti-bacterial and anti-virulence activities of equol (Figure 4 and Figure 5). Equol significantly reduced the expression levels of the carbapenemase genes of *E. coli*, which produce KPC and OXA-48 (Figure 4). More specifically, the expression level of the *bla*_KPC_ gene was more effectively suppressed by equol than that of the *bla*_OXA-48_ gene. However, the result of treatment with meropenem alone showed that the expression level of the carbapenemase gene was slightly decreased. Thus, our results show that equol inhibited the carbapenemase production in *E. coli*.

To investigate the anti-virulence effect, we examined the expression levels of curli and motility-related genes by equol. In the presence of 256 μg/mL equol, the expression level of *csgA*, *csgB,* and *crl* (curli-related genes) in *E. coli* was downregulated by 30.6-, 12.6-, and 11.5-fold, respectively (Figure 5a). As shown in Figure 5b, the motility-related genes were suppressed in a concentration-dependent manner by equol compared to the control. In particular, motility-related genes (*flhD*, *filC,* and *motA*) were significantly downregulated 5.0-, 3.4-, and 3.4-fold, respectively, under the influence of 256 μg/mL equol. These results indicate that equol had an anti-virulence effect by inhibiting curli- and motility-related genes.

## 3. Discussion

The emergence of MDR pathogens is increasing due to the continued use of antimicrobial agents [31]. Especially, resistance to carbapenems, called the last resort of antibiotics, is widespread and has been found in many countries [1,32]. Previous studies have recommended synergistic drug combinations as key strategies for treating MDR pathogens [33,34]. Also, many studies have demonstrated that the development of antibiotic adjuvants that improve antibiotic activity can treat various resistant bacteria infections [35,36,37]. Therefore, antibacterial research and development such as studies on new antibiotic adjuvants should be promoted to prevent the emergence and spread of antibiotic resistance worldwide.

The types of antibiotic adjuvants studied to block antibiotic resistance include *β*-lactamase and efflux pump inhibitors, outer membrane permeabilizers, and anti-virulence drugs [36]. Among them, *β*-lactamase inhibitors are the most successful antibiotic adjuvants used clinically. Also, a previous study reported that anti-virulence drugs were less likely to cause resistant mutations because they do not target essential bacterial components [38]. This study suggested the possibility of equol as a new antibiotic adjuvant through investigations of the synergistic effect of meropenem and equol on carbapenem-resistant *E. coli* strains and the biological activity mechanisms of equol.

First, the MIC results showed that the clinically isolated *E. coli* strains were all resistant to carbapenem antibiotics (Table 1). Yoon et al. reported that CPE detected in Korea from 2011 to 2015 commonly has carbapenemases of KPC, NDM, and OXA-48 [39]. Another study reported that KPC-producing CREs were mainly found in *K. pneumoniae* and *E. coli*, and some *E. coli* also produced OXA-48 [40]. Similar to these studies, our study demonstrated the cause of carbapenem resistance by detecting *bla*_KPC_ and *bla*_OXA-48_ among carbapenemase genes using PCR (Table 2).

A previous study demonstrated that demethyltexasin, a human metabolite of soy isoflavones, showed strong synergistic effects on methicillin-resistant *Staphylococcus aureus* (MRSA) strains when combined with amoxicillin and oxacillin [41]. Likewise, equol is a metabolite of daidzein included in soy isoflavones [22]. Interestingly, previous studies have shown the antimicrobial activity of equol against microorganisms such as *Clostridioides difficile* and *Candida albicans* [29,30]. However, little has been reported about the combined effects of antibiotics and equol. Therefore, this study showed that equol and meropenem exhibited synergistic effects against carbapenem-resistant *E. coli* strains at a FICI of <0.31 using the checkerboard method (Table 3). The combination of equol and meropenem induced the meropenem-resistant *E. coli* strains to be susceptible to meropenem. In addition, the rate and extent of the synergistic effect over time were evaluated through the time killing assay (Figure 1).

This study performed both phenotypic and genotypic analyses to reveal the mechanisms of the synergistic effect of equol combined with meropenem. Biofilm, one of the virulence factors, is a microbial community formed in a self-produced extracellular matrix, which causes resistance to antibiotics [42]. A previous study reported that curli fibers encoded by the *csgBA* operon in *E. coli* were closely related to surface adhesion, cell aggregation, and biofilm formation [43]. In addition, other studies have shown that Crl protein interacted with RpoS and regulated *csgD* [44,45]. Since *csgD* is directly required to activate the *csgBA* promoter, the *crl* gene will affect *csgBA* expression. Consistent with these previous findings, curli-related genes and biofilms showed a tendency to decrease in an equol-concentration-dependent manner (Figure 2). Moreover, our data showed that equol inhibited the expression of *csgB* and *csgA* more than *crl* (Figure 5a). These results indicate that the effect of equol directly influenced the genes encoding curli such as *csgB* and *csgA*.

The organelle responsible for the motility of *E. coli* is the flagellum, which is controlled by the *flhDC* operon [46]. FlhDC activates the transcription of flagella-related genes and several genes such as *fliC* [47]. A previous study reported that the *ΔflhD* and *ΔmotABΔfliC* mutant strains of *E. coli* MG1655 did not show motility [48]. First, our data showed that equol inhibited the motility of carbapenem-resistant *E. coli* at the phenotypic level (Figure 3). Next, the motility-related genes (*flhD*, *filC*, and *motA*) were significantly downgraded by eqoul, which validated the phenotypic effect of equol (Figure 5b). Therefore, this study supported the anti-virulence effects of equol by showing that it inhibits biofilm and motility.

This study also evaluated the transcription levels of the carbapenemase genes by performing qPCR to determine whether the *bla*_KPC_ and *bla*_OXA-48_ genes were reduced by equol alone or in combination with meropenem (Figure 4). The gene expression levels of *bla*_KPC_ and *bla*_OXA-48_ were reduced by equol alone or in combination with meropenem. In contrast, meropenem alone hardly inhibited gene transcription. Particularly, the results of using equol alone or with meropenem significantly reduced the gene transcription level of *bla*_KPC_ compared to control. For the *bla*_OXA-48_ gene, combination therapy with equol and meropenem was more effective, leading to lower gene expression than either treatment alone. In a previous study, equol inhibited the growth and spore formation of *Clostridioides difficile* but had no effect on the expression of toxin-producing genes such as *tcdA*, *tcdB*, *cdtA,* and *cdtB* [29]. However, we observed that equol inhibited the expression of the carbapenemase genes as well as the growth of carbapenem-resistant *E. coli*.

In summary, this study demonstrated the synergistic effect of equol with meropenem on carbapenem-resistant *E. coli*. It also inhibited the expression of specific carbapenemases in *E. coli* as the cause of carbapenem resistance. The anti-virulence effect of equol on *E. coli* was demonstrated through phenotypic and genotypic analyses. Therefore, equol showed the potential as an antibiotic adjuvant and anti-virulence agent. However, further in vivo studies are needed before equol can be used in the pharmaceutical field. Also, additional mechanistic studies are needed to identify the target of equol.

## 4. Materials and Methods

### 4.1. Organisms, Culture Conditions, and Reagents

Two clinical isolates (*E. coli* strains KBN12P05816 and KBN12P06081) were provided by Gyeongsang National University Hospital Branch of the National Culture Collection for Pathogens (GNUH-NCCP). All strains were routinely sub-cultured on MacConkey agar (MAC; Difco, Becton, Dickinson and Company, Sparks, MD, USA) and inoculated into Tryptic Soy Broth (TSB; Difco, Becton, Dickinson, and Company) at 37 °C. The stock cultures were stored in TSB supplemented with 20% glycerol at –80 °C. (S)-Equol used in this study was purchased from TCI, Ltd. (Tokyo, Japan). Carbapenems (meropenem and ertapenem) were obtained from Sigma-Aldrich (St. Louis, MO, USA). (S)-Equol and meropenem were dissolved in DMSO and ertapenem was dissolved in distilled water. The final concentration of DMSO did not exceed 5% in all the experiments.

### 4.2. Minimum Inhibitory Concentration Assay

To determine the MIC of antimicrobial agents (meropenem, imipenem, ertapenem, and equol) against carbapenem-resistant *E. coli* strains, we used the broth microdilution method according to the CLSI guidelines M7-M9 [49,50]. The absorbance of the bacterial suspensions was measured at 600 nm with a Multiskan GO plate reader (Thermo Fisher Scientific, Waltham, MA, USA). The MIC value was defined as the lowest concentration of the antimicrobial agents that inhibited cell growth by 90% compared to the control.

### 4.3. DNA Extraction and Polymerase Chain Reaction (PCR) Amplification

DNA was extracted from the bacterial pellet using a QIAamp DNA Minikit (Qiagen, Valencia, CA, USA) according to the manufacturer’s instructions. The PCR reaction mixture included 5 μL of 10 × PCR buffer (Perkin Elmer, Waltham, MA, USA), 3 μL of MgCl_2_ (1.5 mM; Perkin Elmer), 2.5 μL of dNTPs (2.5 mM; TakaraBio, Shiga, Japan), 5 μL of each primer (10 pmole/μL), 0.4 μL of Ampli Taq Gold (5 U/μL; Perkin Elmer), and 2 μL of extracted DNA in a final volume of 50 μL. The primers used in this study are listed in Table 4. The thermal cycling conditions for PCR amplification consisted of an initial denaturation at 94 °C for 10 min, followed by 36 cycles of denaturation at 94 °C for 30 s, annealing at 55 °C for 30 s, and extension at 72 °C for 50 s, with a final elongation at 72 °C for 5 min. The amplified PCR products were electrophoresed on a 2% agarose gel and visualized using the Gel Doc system (Bio-Rad, Hercules, CA, USA).

### 4.4. Synergy Checkerboard Assay

To assess the antimicrobial combinations, the synergy checkerboard assay was performed in 96-well microtiter plates according to a previous study with reference to the MICs of the two antimicrobial agents [59]. Briefly, bacterial cultures were suspended in TSB at a final concentration of 1 × 10^6^ CFU/mL and distributed in plates with combinations of equol and meropenem using a two-fold serial dilution method. The concentrations of equol and meropenem used were the MIC and sub-MIC concentrations (1/2, 1/4, 1/8, 1/16, and 1/32 × MIC). Plates were incubated at 37 °C for 24 h. Growth inhibition was assessed using a Multiskan GO plate reader, and the FICI for 90% inhibition was calculated as follows: FIC of equol = MIC of equol in combination/MIC of equol alone, and FIC of meropenem = MIC of meropenem in combination/MIC of meropenem alone. The FICI was defined as the FIC of equol plus the FIC of meropenem. The synergistic effect of equol in combination with meropenem against carbapenem-resistant *E. coli* was interpreted as follows. Synergy was indicated by a FICI of ≤0.5, no interaction was indicated by a FICI from 0.5 to 4.0, and antagonism was a FICI of ≥4.0 [60].

### 4.5. Time–Kill Assay

To evaluate the synergistic effect of equol and meropenem against *E. coli*, the time–kill assay was performed using 96-well polystyrene plates according to a previous study [50], with slight modifications. Briefly, *E. coli* suspensions (1 × 10^6^ CFU/mL) in TSB were exposed to antimicrobial agents (1 μg/mL of meropenem, 256 μg/mL of equol, or the combination of 1 μg/mL of meropenem plus 256 μg/mL of equol) and incubated at 37 °C for 24 h. Samples were taken at time intervals of 0, 2, 4, 6, 8, 10, 12, and 24 h and serially diluted in saline. Each sample was inoculated onto Mueller-Hinton agar (MHA; Difco, Becton, Dickinson and Company) plates using a spreader and incubated at 37 °C for 18 h. The number of colony-forming units (CFUs) of the samples was recorded.

### 4.6. Biofilm Inhibition and Eradication Assay

The crystal violet biofilm assay was performed in 96-well polystyrene plates (BD Falcon™, Franklin Lakes, NJ, USA) as previously described [61,62], with minor modifications. Briefly, to determine the effect of equol on biofilm formation, the bacterial cell suspensions were inoculated into TSB containing different concentrations of equol (64 to 256 μg/mL) at a density of 1 × 10^6^ CFU/mL (total volume = 200 μL) and incubated at 37 °C for 24 h. After incubation, the non-adherent cells were removed by washing and fixed at 60 °C for 1 h. The fixed biofilms were stained with 0.5% crystal violet for 10 min. Next, the stained biofilms were solubilized in 250 μL of 33% acetic acid for 20 min and transferred from each well to a new plate. To evaluate the effect of equol on preformed biofilm, the bacterial cell suspensions were inoculated into the plates at a density of 1 × 10^6^ CFU/mL and incubated at 37 °C for 24 h. After incubation, fresh TSB containing various concentrations of equol (64 to 256 μg/mL) was added to each well. After further incubation for 24 h, the biofilm was stained using crystal violet as described above. The total biofilm biomass was quantified by measuring the absorbance at a wavelength of 595 nm using a Multiskan GO plate reader. The sample not treated with equol was used as the control.

### 4.7. Motility Inhibition Assay

The effect of equol on the motility of *E. coli* was determined using Luria Bertani (LB; Difco, Becton, Dickinson and Company) plates containing 0.2% agar as described previously [56,63], with minor modifications. Also, various concentrations of equol were included in the LB agar plates. Briefly, the bacterial cells were grown in TSB at 37 °C for 24 h. Subsequently, 5 μL of bacterial cells (1 × 10^6^ CFU/mL) were inoculated onto LB agar plates and incubated at 37 °C for 24 h. Motility was assessed by measuring the migration distance (mm) excluding the colony diameter after incubation.

### 4.8. RNA Isolation

For the transcriptional analysis of *E. coli*, total RNA was isolated using the following process [62]. Briefly, *E. coli* cells at a density of 1 × 10^6^ CFU/mL in TSB were incubated at 37 °C for 2 h. After incubation, equol and meropenem were added to the bacterial cell suspensions and then further incubated at 37 °C for 8 h. In order to show the effect of the compounds remarkably, equol and meropenem were treated in the exponential phase (Supplementary Materials Figure S1). The cells were collected by centrifuging 2 mL of bacterial suspensions from each sample at 25,000 *g* for 90 s at 4 °C. The total bacterial RNA was isolated and purified from the collected cells using the NucleoSpin RNA Mini Kit (Macherey-Nagel, Düren, Germany). The isolated total RNA was filtered using silica columns and treated with DNase to prevent DNA contamination. The BioDrop µLITE (BioDrop Ltd., Cambridge, UK) was used to measure the quality and concentration of the isolated RNA samples.

### 4.9. Quantitative Polymerase Chain Reaction (qPCR)

QPCR was performed to evaluate the transcription levels of carbapenemase- and virulence-related genes in *E. coli* following equol and meropenem treatment. First, cDNA was synthesized with ReverTraAce qPCR RT Master Mix with gDNA Remover (TOYOBO, Japan) according to the manufacturer’s manual using 1 μg of total RNA. QPCR was performed using TOPreal™ qPCR 2X PreMIX (Enzynomics, Daejeon, Korea) to amplify cDNA in a StepOnePlus Real-Time PCR System (Applied Biosystems, Foster City, CA, USA). The primers used in this study are listed in Table 4. The *16S rRNA* was used as the housekeeping gene. The qPCR conditions consisted of an initial denaturation at 95 °C for 10 min followed by 40 cycles of denaturation at 95 °C of 10 s, annealing at 60 °C for the *16S rRNA* for 15 s, and extension at 72 °C of 30 s. Melting-curve analysis (95 °C for 15 s, 60 °C for 1 min, and 95 °C for 15 s) was added at the end of the qPCR protocol. The annealing temperatures of the carbapenemase- and virulence-related genes are described in Table 4. The transcription levels of the target genes were normalized against those of *16S rRNA* and calculated using the 2^-ΔΔCT^ formula.

### 4.10. Statistical Analysis

The data are presented as means ± standard deviations (SDs). To identify the significant differences between the treated and untreated samples, we used a one-way analysis of variance (ANOVA) followed by Dunnett’s test. The qPCR data were analyzed using the Student’s *t*-test. Also, *16S rRNA* was used as an internal control gene and the relative expression level was analyzed using the 2^-ΔΔ^^CT^ method for the fold change calculation [64]. All statistical analyses were conducted through GraphPad Prism version 5 (GraphPad Software, La Jolla, CA, USA). Statistical significance was considered at * *p* < 0.05, ** *p* < 0.01, and *** *p* < 0.001.

## Figures and Tables

**Table 1:** Minimum inhibitory concentration (MIC in μg/mL) of carbapenems against clinically isolated *E. coli* strains.

Carbapenem	MIC (μg/mL)	
	*E. coli* KBN12P05816	*E. coli* KBN12P06081
Meropenem	16	16
Imipenem	≥4	≥4
Ertapenem	64	32

MIC: minimum inhibitory concentration, KBN: Korea Biobank Network.

**Table 2:** Detection of carbapenemase genes in *E. coli* strains as determined by PCR.

Genes of Carbapenemase	Bacterial Strains	
	*E. coli* KBN12P05816	*E. coli* KBN12P06081
*bla*_KPC_	+	+
*bla*_IMP_	−	−
*bla*_VIM_	−	−
*bla*_NDM_	−	−
*bla*_OXA-48_	+	+

+; denotes the presence of the gene, −; denotes the absence of the gene.

**Table 3:** Combinatory effects of equol and meropenem against carbapenem-resistant *E. coli*.

Strains	MIC (μg/mL)			Meropenem + Equol	
	Meropenem	Equol	Meropenem + Equol	FIC Index	Interpretation
*E. coli* KBN12P05816	16	1024	1 + 256	0.31	S
*E. coli* KBN12P06081	16	1024	1 + 256	0.31	S

FIC: fractional inhibitory concentration, S: susceptible.

**Table 4:** PCR primers used in this study.

Primers	Target Gene	Primer Sequence (5’-3’)	Annealing Temp. (°C)	References
PCR primers	*bla*_KPC_	F: CGTCTAGTTCTGCTGTCTTG	54	[51]
		R: CTTGTCATCCTTGTTAGGCG		
	*bla*_IMP_	F: GGAATAGAGTGGCTTAAYTC	51	[51]
		R: TCGGTTTAAYAAAACAACCACC		
	*bla*_VIM_	F: GATGGTGTTTGGTCGCATA	56	[51]
		R: CGAATGCGCAGCACCAG		
	*bla*_NDM_	F: GGTTTGGCGATCTGGTTTTC	56	[51]
		R: CGGAATGGCTCATCACGATC		
	*bla*_OXA-48_	F: GCGTGGTTAAGGATGAACAC	55	[51]
		R: CATCAAGTTCAACCCAACCG		
qPCR primers	*bla*_KPC_	F: GATACCACGTTCCGTCTGG	57	[52]
		R: GCAGGTTCCGGTTTTGTCTC		
	*bla*_OXA-48_	F: GGCACGTATGAGCAAGATGC	59	[53]
		R: GTTTGACAATACGCTGGCTGC		
	*csgA*	F: GGGCTCAGATGACAGCTCAAT	59	[54]
		R: GCCGTTCCACTGATCAAGAGTAG		
	*csgB*	F: CATAATTGGTCAAGCTGGGACTAA	55	[54]
		R: GCAACAACCGCCAAAAGTTT		
	*crl*	F: TTTCGATTGTCTGGCTGTATG	54	[55]
		R: CTTCAGATTCAGCGTCGTC		
	*motA*	F: ACAGGTAGCGCGTTCTCACT	58	[54]
		R: AGCGTGGATAAACCGATACG		
	*flhD*	F: ACTTGCACAGCGTCTGATTG	55	[56]
		R: AGCTTAACCATTTGCGGAAG		
	*fliC*	F: ACAGCCTCTCGCTGATCACTCAAA	61	[57]
		R: GCGCTGTTAATACGCAAGCCAGAA		
	*16SrRNA*	F: CAGCTCGTGTCGTGAGATGT	60	[58]
		R: CGTAAGGGCCATGATGACTT		

## Data Availability

All data are presented in the manuscript.

## Supplementary Materials

The following are available online at https://www.mdpi.com/2079-6382/10/2/161/s1, Figure S1.
