# Supplementary figures and images for: Synergistic Activity of Equol and Meropenem against Carbapenem-Resistant Escherichia coli
Source: Antibiotics (Basel). 2021 Feb 5;10(2):161. doi: 10.3390/antibiotics10020161 (PMC7914716; doi:10.3390/antibiotics10020161)

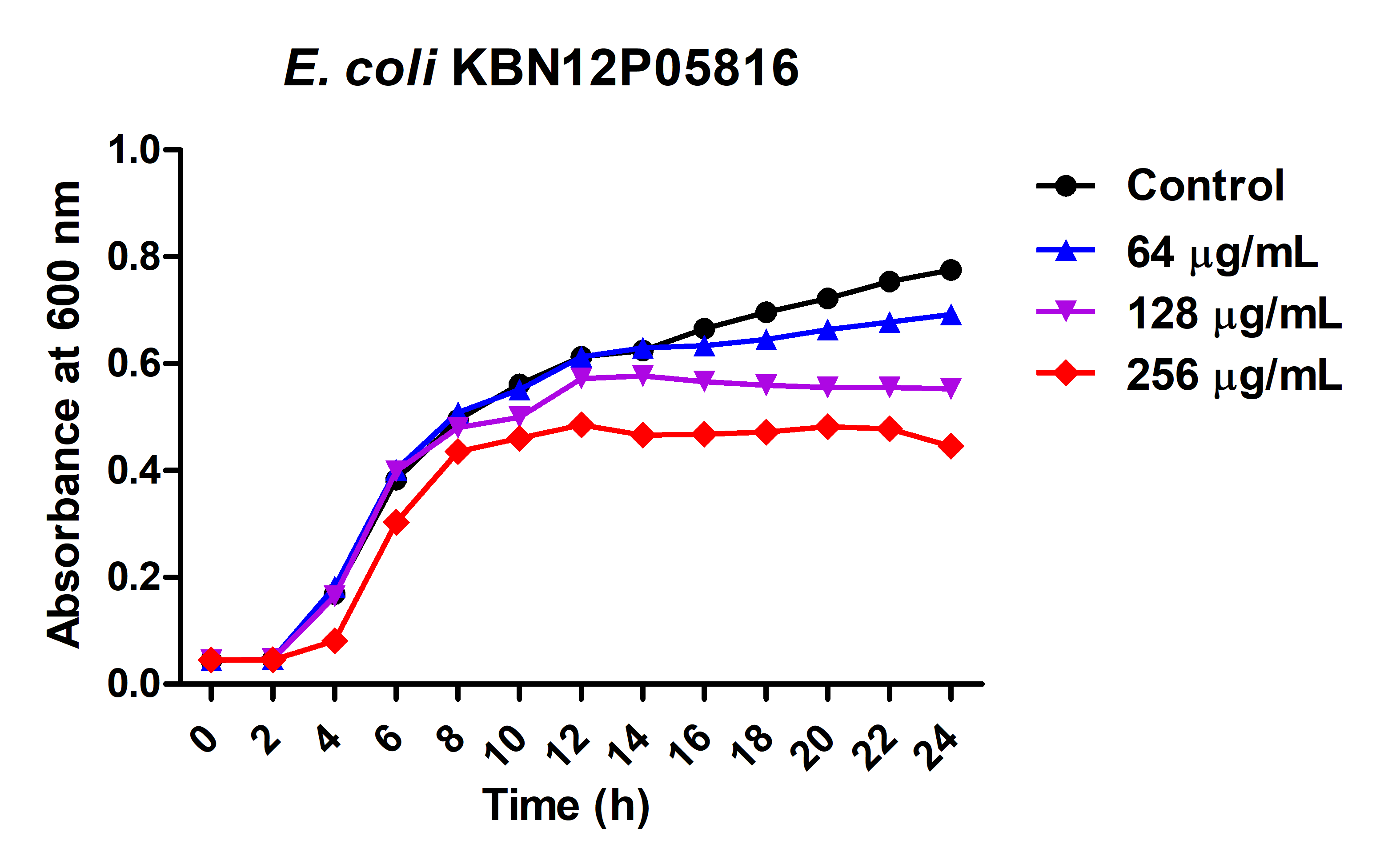

Supplement: Supplementary file 1 [file antibiotics-10-00161-s001.zip › antibiotics-1094574-supplementary.tif]
